# Interference Competition and High Temperatures Reduce the Virulence of Fig Wasps and Stabilize a Fig-Wasp Mutualism

**DOI:** 10.1371/journal.pone.0007802

**Published:** 2009-11-12

**Authors:** Rui-Wu Wang, Jo Ridley, Bao-Fa Sun, Qi Zheng, Derek W. Dunn, James Cook, Lei Shi, Ya-Ping Zhang, Douglas W. Yu

**Affiliations:** 1 Ecology, Conservation, and Environment Center (ECEC), State Key Laboratory of Genetic Resources and Evolution, Kunming Institute of Zoology, Chinese Academy of Sciences, Kunming, Yunnan, China; 2 Centre for Ecology, Evolution and Conservation (CEEC) and School of Biological Sciences, University of East Anglia, Norwich, Norfolk, United Kingdom; 3 Key Laboratory of Zoological Systematics and Evolution, Institute of Zoology, Chinese Academy of Sciences, Beijing, China; 4 School of Medicine, Zhejiang University, Hangzhou, Zhejiang, China; 5 School of Biological Sciences, University of Reading, Reading, Berks, United Kingdom; 6 Statistics and Mathematics College, Yunnan University of Finance and Economics, Kunming, Yunnan, China; University of Arizona, United States of America

## Abstract

Fig trees are pollinated by fig wasps, which also oviposit in female flowers. The wasp larvae gall and eat developing seeds. Although fig trees benefit from allowing wasps to oviposit, because the wasp offspring disperse pollen, figs must prevent wasps from ovipositing in all flowers, or seed production would cease, and the mutualism would go extinct. In *Ficus racemosa*, we find that syconia (‘figs’) that have few foundresses (ovipositing wasps) are underexploited in the summer (few seeds, few galls, many empty ovules) and are overexploited in the winter (few seeds, many galls, few empty ovules). Conversely, syconia with many foundresses produce intermediate numbers of galls and seeds, regardless of season. We use experiments to explain these patterns, and thus, to explain how this mutualism is maintained. In the hot summer, wasps suffer short lifespans and therefore fail to oviposit in many flowers. In contrast, cooler temperatures in the winter permit longer wasp lifespans, which in turn allows most flowers to be exploited by the wasps. However, even in winter, only in syconia that happen to have few foundresses are most flowers turned into galls. In syconia with higher numbers of foundresses, interference competition reduces foundress lifespans, which reduces the proportion of flowers that are galled. We further show that syconia encourage the entry of multiple foundresses by delaying ostiole closure. Taken together, these factors allow fig trees to reduce galling in the wasp-benign winter and boost galling (and pollination) in the wasp-stressing summer. Interference competition has been shown to reduce virulence in pathogenic bacteria. Our results show that interference also maintains cooperation in a classic, cooperative symbiosis, thus linking theories of virulence and mutualism. More generally, our results reveal how frequency-dependent population regulation can occur in the fig-wasp mutualism, and how a host species can ‘set the rules of the game’ to ensure mutualistic behavior in its symbionts.

## Introduction

Most organisms play host to a variety of beneficial smaller organisms. For example, species as diverse as humans [1], plants [2], [3], and insects [4] use symbionts to augment their diets and to protect themselves against parasites. Despite their prevalence, explaining how host-symbiont relationships remain mutualistic is a major challenge. Whenever two species interact, their interests are never exactly aligned. As a consequence, mutualisms only persist to the extent that each party gains more by investing in the other partner than it would by investing in itself [5], [6]. In the case of host-symbiont relationships, the mutualism is additionally unstable because each host has many symbionts. A symbiont making a short-term sacrifice to benefit the host will also indirectly benefit all the other symbionts in the same host, which are its competitors [6], [7].

Although the conflict between host and symbiont is traditionally presented in terms of symbionts choosing whether or not to cheat on their passive hosts, a recent focus has been on how hosts use mechanisms to encourage mutualists [1] and constrain pathogens [8] by targeting investment preferentially at mutualists [3], [7], [9]–[14] or even by directing the evolution of symbionts [15]–[20]. How hosts favor mutualistic over parasitic symbionts is now seen to be fundamental to subjects as disparate as medicine [1] and ecosystem services [21].

One important example of a host-symbiont mutualism is when plants receive pollination services from insects that in return obtain benefits from the plants. The most common benefits are a simple reward of pollen or nectar to the pollinating adult insect, but the reward can also be a sacrifice of some plant reproductive tissue for consumption by pollinator larvae [18], [22]. A long-studied, indeed classic, example of such a system is the symbiosis between fig plants (*Ficus* spp.) and their pollinator wasps (*Agaonidae*) [19], [20], [23]–[28]. The fig-pollinator mutualism has persisted for >60 million years and radiated into >750 *Ficus* species, with associated wasps [29], [30]. Fig wasps pollinate fig flowers (within the urn-like inflorescences that are technically known as syconia and colloquially known as ‘figs’), but also lay their eggs in viable ovules.

Crucially, each uni-ovulate flower receives only a single egg, and thus, each larva galls and then consumes a potential fig seed. Both wasp and seed production benefit the fig host, since wasp offspring carry pollen to other trees, but only wasp production benefits the wasps; seeds represent foregone wasp fitness. Thus, selection should favor wasps that successfully convert more fig ovules to offspring, with the long-term outcome being that seed production will cease. Because each species of fig wasp exploits only one fig plant species [24], [26], [31]–[34], wasp extinction would follow the extinction of its host. The persistence of each fig-wasp mutualism thus requires a mechanism that guarantees the persistence of seed production, despite the short-term costs to individual wasps.

For approximately half of fig species, the conflict between wasp and seed production is resolved via gynodioecy. ‘Male’ trees produce syconia in which all ovules receive wasp eggs, and these trees only produce wasps. ‘Female’ trees produce syconia in which no ovules receive wasp eggs, because the floral styles are too long for wasp ovipositors to reach the ovules [35]. These trees only produce seeds. Dispersing wasp foundresses fail to avoid female figs, despite a zero expected fitness, because of a sensory trap [36]. Male and female figs evolve the same bouquet of volatile chemicals [37], [38], thus making it impossible (or almost impossible [39]) for female wasps to identify the sex of a tree.

For monoecious fig species, the syconia of which produce both seeds and wasps, explaining how the wasp-seed conflict is resolved is more challenging [reviewed in ref. 20]. For instance, fig trees do not selectively abort over-exploited syconia [40], and dispersal limitation of wasps [41] does not explain seed production in the many syconia that receive sufficient numbers of wasp foundresses to oviposit in all ovules [20], [25], [28], [32], [42].

Mutualism stability is instead thought to arise somehow from the highly variable lengths of floral styles within the syconia of monoecious *Ficus* [20], [43]. Variable style lengths may function specifically to prevent wasps from overexploiting fig trees [35], [43]. This is because ovipositing wasps (foundresses) prefer shorter styled inner ovules [19], [44]–[46], even though they are able to oviposit in all or most of the long-styled outer ovules [19], [41]. Several hypotheses exist in the literature to explain why outer ovules are unattractive to foundresses. These include: being slow to oviposit into [20], reducing the probability that female offspring get released by males [47], and being more prone to parasitism [19]. However, although these mechanisms explain selection to oviposit preferentially in inner ovules, they do not explain why wasps in multi-foundress syconia cannot, or do not, ultimately also lay eggs in the outer ovules [28].

The simplest answer is that foundress wasps' lives are too short to fully exploit all ovules, leaving outer ovules to develop as seeds. A simple extrapolation, assuming that *n* wasps can oviposit for *n* times as long as a single wasp, still means that in many cases, small groups of wasps have enough combined lifespan to exploit all ovules in a syconium [20]. This is true even if wasps switch to outer ovules only after every inner ovule contains a wasp egg.

However, the combined lifespans of all wasps could nonetheless turn out to be too short to allow the total exploitation of outer ovules if: (1) foundresses reduce each others' oviposition rates (*n* foundresses oviposit less than *n* times as long as one foundress) and/or (2) environmental stresses shorten wasp lifespans at least some of the time. Under the first option, wasps could interfere with each others' egg laying directly (e.g. by fighting or by impeding each other [48] or indirectly (e.g. by depleting the oxygen supply). Under the second option, it is known that wasp lifetimes are shorter when humidity is low [47] and the ambient temperature is high [49].

In this paper, we test these possibilities by using a combination of manipulative experiments and longer-term survey data from the fig species *Ficus racemosa* L. We test three specific hypotheses. (1) Foundresses oviposit optimally: they lay in outer ovules only as inner ovules fill up; (2) there is interference among foundresses: *n* foundresses oviposit for less than *n*-times as long as one foundress; and (3) seasonal variation in wasp survivorship explains seasonal variation in oviposition levels. We then use these results to develop an adaptive explanation for a control mechanism that is new to the *Ficus* literature: the density-dependent closure of the opening (ostiole) in the receptive syconium, through which wasps enter.

## Results

### Environmental effects on foundress lifespans

Previous work has found that the seed:wasp ratio in *F. racemosa* varies with season [50]. Hence, we first require a non-arbitrary classification of ‘season’. To do this we fit a model of mean expected lifespan to our laboratory flask experiment (**Methods: Environmental effects on foundress lifespans**). *Ceratosolen fusciceps* wasps have shorter lifespans in warmer and dryer conditions, with Mean lifetime  = 4.0−0.13×Temp + (0.017+0.0004×Temp) × Humidity. All terms are significant at p<0.001.

This model is then combined with daily data for average relative humidity and average temperature from July 2004 to June 2007, downloaded from http://www.wunderground.com/history/station/56959/2006/6/1/MonthlyHistory.html#calendar (accessed 1 July 2008). These data were pooled across years to yield monthly means, which were transformed to lifespan estimates using the model fitted to the laboratory data (Figure S3), and we then grouped the months into two seasons by minimizing the sum of the within-season coefficients of variation in lifespans: Min[CV(Summer lifespans) + CV(Winter lifespans)]. This results in November, December and January being designated as ‘winter’, and the remaining nine months as ‘summer’.

### Seasonal variation in galling and pollination

**Figure 1:**
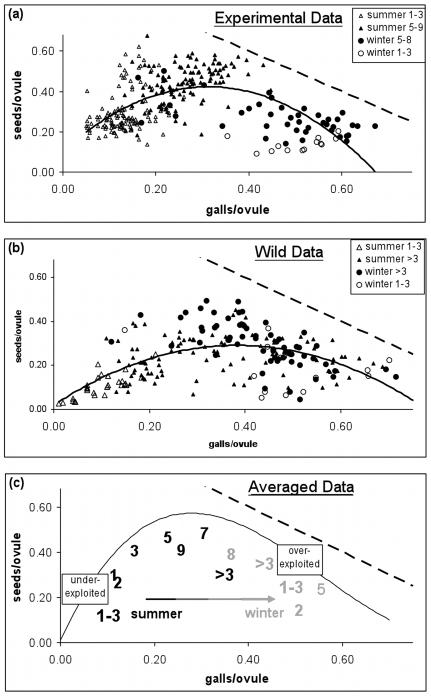
Relationships between galling and pollination levels. (A) Syconia in which foundresses had been introduced experimentally, (B) wild-collected syconia, and (C) the same data re-plotted showing only the means. The dashed lines on each figure (upper left to lower right) indicate 100% pollination. Experimental introduction syconia are plotted by exact foundress number, while wild data are shown as 1–3 foundresses or >3 foundresses. Points in the lower left quadrant indicate a wasted resource: empty ovules. The wasp's interests are served only by galls, so wasps are selected to achieve points toward the lower right, which occurs in the winter, when lifespans are longer. Increasing foundress number tends to lead to intermediate numbers of seeds and galls in both seasons (statistical analyses in text). Legend: Hot refers to summer months, Cold to winter months.

Using this classification of seasons, we can use generic statistical models to make inferences about total pollination level (seeds + galls) and the nature of the seed-wasp trade-off (Figure 1).

In the summer, syconia with more foundresses leave fewer ovules unpollinated (linear regression model: %vacant ovules  =  foundress number, wild data: t = −3.3, n = 219, p = 0.001, β = −0.004, R^2^ = 7.2%; experimental data: t = −15.6, n = 241, p<0.001, β = −0.047, R^2^ = 50.4%). In the winter, foundress number does not significantly affect the proportion of vacant ovules (wild data: p = 0.68, β  = 0.0004, R^2^ = 0.2%; experimental data: p = 0.76, β = 1.9×10^−7^, R^2^ = 0.2%). This can be seen easily in Figure 1c: summer data points with more foundresses tend to be closer to the dashed line indicating complete pollination of ovules, whereas all the winter data points lie near to and roughly parallel to that line.

In addition, the nature of the seed:wasp tradeoff also appears to change across season (statistical details in Text S1). In the summer, the proportion of pollinated flowers that receive a wasp egg (galls/(galls+seeds)) either increases with or does not change with the number of foundresses (Random effects GLM; experimental data [Figure 1a], Foundress number × Season_Summer_ interaction effect, β = 0.0119±0.0016SE, df = 312, t = 7.33, p<0.001; wild data [Figure 1b], β = 0.0001±0.0007SE, df = 210, t = 0.15, p = 0.88). In other words, in the summer, adding more foundresses never decreases the proportion of pollinated ovules that also receive a wasp egg (Figure 1c).

In the winter, the picture is more complex and surprising. The wild, winter dataset is consistent with the summer dataset in that adding more foundresses significantly increases the proportion of galled ovules (Figure 1c, β = 0.0032±0.0011SE, df = 210, t = 2.82, p = 0.005). However, in the experimental, winter dataset, increasing the number of foundresses significantly *decreases* the proportion of galled ovules (Figure 1c, β = −0.0448±0.0043SE, df = 312, t = −10.33, p<0.001). In other words, in the winter, adding more foundresses can decrease the total number of eggs laid and eliminate the seed:wasp tradeoff, at least under experimental conditions, where foundresses were introduced consecutively. Moreover, even in the wild dataset, note that the positions of the centroids of the low- vs. high-foundress syconia (1–3 vs. >3, Figure 1c) suggest an overall negative effect of foundress number on galling proportion.

Another way of interpreting Figure 1 is to note that we can decompose the interaction of season and seed:wasp ratio into two separate effects: (i) syconia produce relatively more galls in the winter than they do in the summer [50], and (ii) this seasonal difference in galling is more pronounced in few-foundress syconia than it is in many-foundress syconia. Most importantly, regardless of season, syconia with many foundresses tend to exhibit intermediate proportions of seeds and galls (Figure 1c).

### Variation in ovule selectivity and oviposition lifespans

The above analysis uses a general linear model to test for significant effects of foundress number and season on the fig-wasp conflict. The advantage and disadvantage of such an approach is that it is free of mechanism. Thus, while we now have some statistical indication that lower temperatures increase galling success and that the effect of foundress number on galling success can reverse between seasons, we lack a mechanistic explanation. In short, the data in Figure 1 so far have only provided us with a series of observations to explain, albeit observations in which we have some confidence after statistical analysis.

In order to understand how variation in season and foundress number produces the observed patterns in galling and pollination (Figure 1), we need to quantify what it is about oviposition behavior that changes. We therefore turn to a previously published model of oviposition behavior in fig wasps [20] to convert the currencies of galls and seeds into the currencies of oviposition lifespan and ovule selectivity (Figure S1). That is, we use a functional model, not a statistical model, to infer from the seed and gall data (Figure 1) how many ovules foundresses probed during their lives and which ovules they chose.

**Figure 2:**
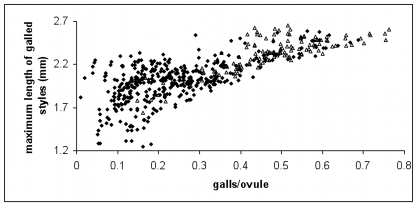
Model-estimated maximum accepted style lengths across all galled ovules in both experimental and wild datasets, in the summer (♦) and the winter (▵). Style lengths are higher in syconia with more galled ovules, but the relationship between style lengths and galls/ovule is invariant across seasons (linear model, max_style  =  (1.26×%galls) + (0.0040×season)−(0.076×%galls:season), p-values: <0.001, 0.95, 0.58 respectively, R^2^ with season  = 0.565, R^2^ without season  = 0.565. Accordingly, differences in oviposition behavior do not explain seasonal differences in the relationship between galls and seeds.

Our best-fit oviposition model produces a single curvilinear relationship between percentage galling and the maximum style length accepted for galled ovules that fits both the wild and experimental datasets (Figure 2). This is consistent with optimal foraging theory and with empirical data [19], [20], which suggest that foundresses have evolved to accept increasingly longer styles as shorter-styled ovules fill up. However, because this decreasing selectivity does not appear to vary with either season or foundress number, we conclude that variation in ovule selectivity is not the mechanism that produces the patterns in Figure 1.

**Figure 3:**
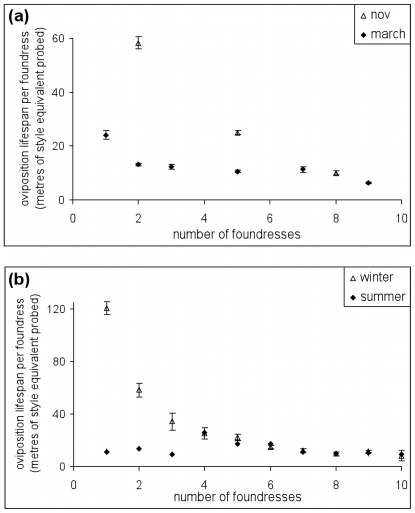
Oviposition lifespans for foundresses in different seasons, averaged across syconia. Model output for simultaneous foundress introductions in (A), and for wild syconia in (B). For syconia with many foundresses, oviposition lifespans are similar, irrespective of season, but for syconia with a few foundresses, oviposition lifespans are shorter in the summer (March).

Instead, our oviposition model indicates that variation in foundress lifespans is responsible for the observed variation in the seed:wasp ratio (Figure 1). Estimated working lifespans decrease as the number of foundresses increases (Figure 3). Also, estimated lifespans are longer in cold months (consistent with the lifespan experiment), but only when foundress numbers are low (Figure 3). Thus, our oviposition model suggests two, interacting factors that reduce the time available to foundresses for oviposition: high ambient temperatures and the presence of other foundresses.

**Figure 4:**
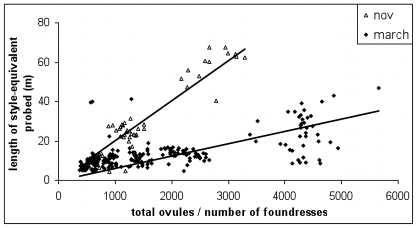
Model-estimates of foundress lifespan for the experimental data, plotted against resource availability (total ovules in each fig divided by the number of foundresses) in the summer (March, ♦), and the winter (November, ◊). Because zero ovules implies zero probed styles, both regression lines are forced through the origin. The slopes for both regressions are significant at p<0.001, winter R^2^ = 77.8%, summer R^2^ = 95.9%.

These two patterns can be seen clearly by plotting estimated lifespan against resource availability (ovules per foundress) (Figure 4). Two clear lines emerge, one for each season, with resource availability explaining a large proportion of variation in oviposition rates. Such relationships between resource use and resource availability are the hallmark of interference competition [51], [52], which is frequently modeled using the Hassell and Varley [53] model, here taking the form
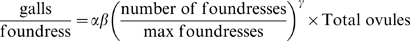
(1)


*α* is a unit-less, season-dependent scaling parameter, and *β* is a scaling parameter governing the fraction of galled ovules when there is only one foundress (galls/ovule/foundress). *γ* governs the strength of the relationship between oviposition rate and foundress number, and if negative, indicates interference competition. Max foundresses  = 9 in this case, being the largest number of introduced foundresses. *α* can be equated with season-dependent lifespans: 11 (hours) in March and 20 (hours) in November (Figure S3). If we use the Solver function from Microsoft Excel 2002 to minimize the sum of squares difference by adjusting *β* and *γ*, Eqn. 1 explains 72.3% of all variation in galling for the 287 consecutive-introduction syconia (*β* = 0.04 and *γ*  = −0.61). The negative value for *γ* indicates that adding foundresses decreases per-foundress fecundity. A re-sampling test (with replacement, 1000 iterations) finds that *γ* is significantly less than 0 (min  = −0.74, max  = −0.51, sd = 0.035).

If we instead fit all three parameters to the data (resulting in *α_summer_* = 1, *α_winter_* = 3.72, *β* = 0.02 and *γ* = −0.90), we explain 84.2% of variation in galling. This second set of parameters indicates that survival is 3.72 times higher in November than it is in March. That is, relative to the lifespan experiments, seasonal variation in lifespans appears to be magnified if measured inside the lumen (i.e. 20/11 is only 1.85). The fact that Eqn. 1 can explain over three-quarters of variation in galling success, measured across syconia in different seasons, with different numbers of ovules and different numbers of foundresses, suggests that it closely models real-life oviposition and therefore that interference competition occurs among foundresses.

### Experimental evidence that wasps reduce each others' oviposition rates

Thus far, we have observed gall and seed data which, through the medium of an oviposition model, imply that as the number of foundresses in a syconium increases, the effective lifespan of each is reduced (Figure 3). Here, we support this signal of interference competition with an experimental result.

**Figure 5:**
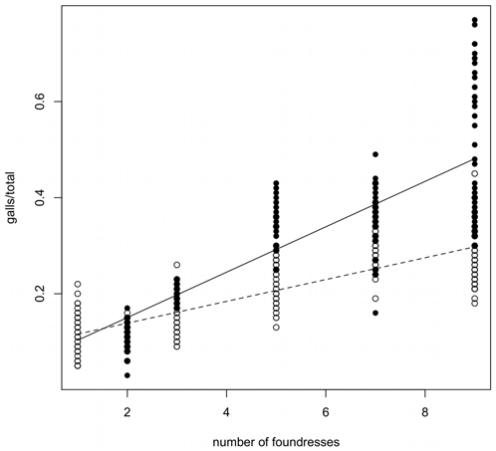
Galling data under consecutive (○) and staggered (•) introduction experiments. As more live foundresses co-occur in the syconium, galling rates are depressed. This confirms the theoretical result from our oviposition model and is consistent with interference among foundresses. Fitted lines are linear regressions (dashed  =  consecutive, solid  =  staggered).

When wasps are experimentally introduced over 33 hours such that the temporal overlap of ovipositing foundresses is reduced (the staggered-introduction treatment), galling rates are significantly higher than in the consecutive-introduction treatment (all foundresses introduced within 30 mins), which maximizes temporal overlap (Figure 5). The Treatment × Foundress number interaction effect is significantly positive, meaning that the effect is stronger as more foundresses are introduced (general linear mixed model, with tree included as a random factor; β_Foundress number_ = 0.019, p<0.0001, β_staggered treatment_ = −0.032, p<0.0001, β_Foundress×Staggered treatment_ = 0.024, p<0.001, statistical details in Text S2). A random-factor model without the interaction effect has higher AIC and BIC values, so we retain the interaction. A fixed-effects model with tree included as a categorical factor produces similar results (S5), with an R^2^ of 77.0%. Foundresses thus do lay fewer eggs when they are in the company of other live foundresses.

### Experimental evidence that lifespans are shorter at higher wasp densities and temperatures

Previously, we found that wasp lifespans are shorter when ambient temperatures are higher and humidity is lower (**Results** **: Environmental effects on foundress lifespans**), which implies seasonal variation in wasp lifespans (Figure S3) and which can explain reduced galling rates in the hot months (Figures 1, 4). We now extend our analysis by taking advantage of the fact that the starting densities of wasps in the flask experiment varied between 28 and 232 across the experimental replicates (as a consequence of different densities of wasps in collected syconia). If we add the starting wasp density in each flask as a third explanatory variable, temperature, humidity, and starting density all significantly affect survival (Cox proportional hazard model, mean mortality  = 0.40×temp–8.62×humidity + 0.018×density–0.00064×temp × density, all four terms significant at p<0.001; z = 33.0, −58.2, 7.5, −7.3 respectively). Thus, in the laboratory experiment, starting density significantly increases mortality, and this effect weakens as temperature increases.

The negative effect of starting density on lifespan is consistent with the observation that foundresses in multi-foundress syconia are estimated to spend less time ovipositing than are foundresses in few-foundress syconia (Figure 3). The negative coefficient for the temperature × density interaction is also consistent with the observation that effective lifespan declines less steeply with foundress number in the summer, simply because lifespans in the summer are never that long (Figure 3).

### Host control via density-dependent ostiole closure

**Figure 6:**
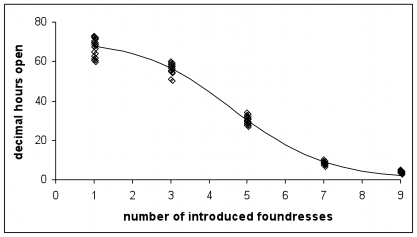
Ostiole closure in syconia with different numbers of simultaneously introduced foundresses. With no introduced foundresses, the ostiole remained open for 474 hours (n = 8). The ostiole closes more quickly when more foundresses are introduced (logistic regression, p<0.001, n = 55, variance explained  = 97.5%).

We have observed that syconia are underexploited in the summer (Figure 1). We next present empirical evidence suggesting that fig trees profit from this situation to minimize vacant ovules whilst maximizing seeds per gall. The experimental introduction of wasps shows that the ostiole stays open longer when fewer wasps have been introduced (Figure 6). It is easy to suppose that density-dependent ostiole closure prevents the entry of too many foundresses, but our simulations (Methods: **Estimating the effect of ostiole closure on foundress number distributions**) suggest instead that it is more effective at reducing the frequency of syconia that receive only a few foundresses.

In the winter, without density-dependent ostiole closure, 25% of figs would receive only 1–3 foundresses, as compared with the observed figure of 17%. Similarly, in the summer, 18% of syconia would receive 1–3 foundresses, instead of the observed 8%.

Multiplying these foundress number distributions by the observed galling and pollination rates recorded in the wild suggests that ostiole closure increases galls by 8% and seeds by 6% in the summer, and increases seeds by 4% for a 1% drop in galls in the winter. In summary, by maintaining an open ostiole for longer when initial foundress entry rate is low, pollen limitation caused by too few entering wasps is made less likely. We note that the experiment was conducted in November-December 2001, so we are assuming that ostiole closure behavior is similar across seasons. Preliminary trials conducted in the summer (April-May 2006) have found indistinguishable results (R. Wang, unpublished data).

## Discussion

A generic statistical analysis of wild-collected and experimental fig data (Figure 1) suggested that foundresses achieve higher galling success in colder weather and that galling rates can decrease with foundress number in the winter. The latter effect helps to ensure seed production in figs. To investigate these observations more rigorously, we used a mechanistic oviposition model (Figure 2) and inferred that foundress effective lifespan is lower at higher ambient temperatures (consistent with our experimental results) and when in the company of other live foundresses (Figures 3 and 4), which is suggestive of interference competition. We then provide statistical (Eqn. 1) and experimental evidence (Figure 5, ***Results*** ***: Experimental evidence that lifespans are shorter at higher wasp densities and temperatures***) consistent with interference competition amongst pollinator wasps in *Ficus racemosa*.

The seed and gall data (Figure 1) are strongly suggestive of optimal foraging behavior in ovipositing fig wasps: foundresses accept outer ovule styles after inner ovules become exhausted, which in turn contributes to stability in the fig-pollinator mutualism [20] (Figure 2). We start by noting that at the highest foundress numbers, seeds outnumber galls, especially in the summer (Figure 1). Style fusions arise such that the styles of ovules close to the centre of the syconium are typically each fused to more than one longer style, while long styles are fused to only one shorter style. Consequently, ovipositing only in inner ovules leads to more seeds/gall than does ovipositing indiscriminately. Since we find more seeds per gall in syconia with fewer galls (points tend to be above the 1∶1 line on the left hand side of Figure 1, especially in the experimental dataset), this suggests that wasps gall inner ovules before outer ovules (Figure 2). Inner ovules are more profitable for foundresses [19], [20], [47], and a separate study has documented that oviposition is concentrated in inner ovules in an Australian population of *Ficus racemosa* [19].

The unimodal relationship between gall and seed production (Figure 1) can also be used to reveal mechanisms that promote stability in this fig-wasp system. Starting from the lower left of Figure 1c, as galling increases, seeds also increase. In *F. racemosa*, wasps actively deposit pollen during oviposition [54], and because multiple floral styles are fused together (see **Methods**), this triggers multiple pollinations for each oviposition event. In this quadrant of Figure 1, the wasps are purely beneficial to the tree, converting empty ovules to seeds and wasps. In the upper centre of Figure 1c, some 80–90% of ovules are pollinated (30–40% galled, 50–60% seeds). Finally, moving towards the bottom right of Figure 1c, we observe that an increasing proportion of those pollinated ovules are galled, revealing the trade-off between wasp and seed production.

Figure 1 thus indicates that the strength of conflict between *F. racemosa* and its wasps varies across seasons [50]. In the summer, wasp lifespans are so short (Figure 3) that the challenge for the figs is to increase wasp numbers so that ovules are not left empty. There is no conflict. In contrast, in the winter, wasp lifespans are long enough for full ovule exploitation so that even a few foundresses can oviposit in most ovules, and conflict between fig and wasp is high. Thus, in the winter, the challenge for the fig is also to increase foundress numbers, but this time, in order to intensify interference competition (Figure 5) and, thus, to reduce wasp lifespans (Figure 3).

One way that *F. racemosa* meets the challenge of increasing foundress numbers is to exhibit density-dependent closure of the ostiole (Figure 6), which appears to reduce the proportion of syconia with low foundress numbers. This is consistent with the interpretation that a high proportion of galls to seeds is not in the fig's interest. We further observe that (**Methods: Estimating the effect of ostiole closure**) mean foundress number per syconium is lower in the winter. This may simply be due to environmental factors: lower success in locating figs or higher syconium availability. However, if this is due to the fig's control, we might also infer that *F. racemosa*'s optimal offspring ratio skews towards wasps in the winter. There is also evidence for possible density dependent ostiole closure in two distantly related *Ficus* species, *F. aurea* and *F. carica* [55]. More generally, the ability to regulate foundress numbers and/or competition may be a reason why fig trees evolved enclosed inflorescences [18]. However, an alternative explanation for the origin of ostiole closure is that it protected seeds and wasps from predators [40].

From the perspective of mutualism stability in *F. racemosa*, the key result is that increasing the number of foundresses in a syconium tends to result in more seed production, guaranteeing the persistence of the symbiosis (Figures 1 and 5). Inference from raw seed and gall data (Figures 3 and 4) and direct experimental support (Figure 5 and density effects in the laboratory lifespan data) all suggest that the presence of other foundresses reduces total oviposition, especially in the winter when baseline lifespans are long (Figure 3). Furthermore, a standard interference model, drawn from behavioral ecology [53], explains around three quarters of all variation in galling rates for consecutive-introduction syconia, despite the fact the dataset includes syconia from different seasons and with differing numbers of ovules. Interestingly, Vigneux et al. [56] have shown that interference competition amongst pathogenic bacteria can reduce virulence, suggesting a fundamental link between theories of virulence and mutualism.

What is the mechanism behind interference competition? Given that lifespan decreases with ambient temperature, a straightforward explanation is that increased wasp density also results in increased ambient temperatures and, consequently, in reduced lifespans (Figures 3, 5). This might be caused by a build-up of metabolic heat. For example, air temperatures inside the tents of tent caterpillars rise by up to 6.5°C above ambient temperature [57]. However, a simple model of heat production by fig wasps suggests they will have almost no effect on the temperature inside the lumen (Text S3). Hence, while direct heating appears unlikely, it remains possible that close proximity exacts a physiological toll on fig wasps indirectly, perhaps due to an increase in activity in the presence of competitors. Foundresses might even fight with each other [48]. If the wasps do indeed overheat at higher densities, because of their physical proximity or higher activity, the closing of the ostiole will tend to exacerbate this.

The negative effect of wasp density on lifespans could also be due to *F. racemosa* itself. The syconium might monitor the number of pollen tubes being formed and regulate the temperature inside accordingly, perhaps by regulating transpiration. For instance, Patiño et al. [58] found that in Neotropical *Ficus* with large- and medium-size mature syconia, transpiration prevents the developing wasps within from overheating. However, because wasps find it individually profitable to pollinate the ovules into which they oviposit, since offspring survivorship is increased [59], the combined force of pollination reveals information that the plant can use to monitor the system. It remains to be established why this mechanism would work less well in the winter such that we observe a higher proportion of ovules galled (Figure 1), although colder ambient temperatures should naturally reduce the rate at which figs heat up after transpiration is cut off.

Whatever the mechanistic basis to competition among foundresses, its consequence is that if wasp population density increases relative to that of *F. racemosa* trees, more seeds and fewer galls will be produced per syconium. This will lead to more figs and fewer wasps, and thus, to co-regulation of the two populations. Such frequency-dependent population regulation is essential for stability in interacting species [60], but we are unaware of any co-regulation mechanisms having been documented in any mutualism.

### The interaction with Local Mate Competition

In all fig species investigated to date, pollinator male sex ratio increases with foundress number [61], as predicted by Local Mate Competition (LMC) theory [62], [63]. The exact quantitative predictions of LMC theory also depend on other variables such as variation in foundress brood size and levels of inbreeding, which require genetic estimation [e.g. 64]. However, foundress number is the main driver of observed sex ratios, which change from a typical value of about 10% male with n = 1, to 25% with n = 2, and 40% with n = 4. As foundress number increases further, the sex ratio asymptotes around 50%. There is evidence that the mechanism behind these changes is the decreased brood size of multiple foundresses, combined with the fact that females lay most male eggs early on [65], which is consistent with our finding (Figure 3) that effective lifespan decreases with foundress number. Male wasps do not disperse pollen, although some are needed to mate with and to release females from both their galls and the syconia. Consequently, if we consider the effect of LMC in isolation, wasps may serve fig interests better in species that typically have low foundress numbers [25].

However, in *Ficus racemosa*, LMC appears to have, at most, only minor effects on the benefits to the fig of increased foundress number. Preliminary data (R.W. Wang, B.F. Sun, unpublished data) reveal that male sex ratio increases from a mean of 17.3% (at n = 2) to an apparent asymptote of ≈30% (at n = 5, 7, 9), which is a relatively small LMC effect and suggests the presence of countervailing factors. In contrast, in the summer, increasing foundress number increases both seeds and wasp offspring by several times (Figure 1). And in the winter, because the effect of increasing foundresses is to decrease the proportion of galls (Figure 1C), the increasing male sex ratio is multiplied against a decreasing number of galls, resulting in approximately the same absolute number of male offspring (Figure 1c). Thus, at n = 2 foundresses, the gall proportion (≈50%) multiplied by the male sex ratio (17.3%) results in ≈8.7% male wasps out of all galls and seeds. At n = 8, the same calculation (≈0.35 galls x ≈0.30 male) results in ≈10.5% male wasps.

### Summary and generality

In summary, temperature and foundress number contribute to variance in fig wasp lifespans, and lifespan determines variation in galling, which determines variation in seed production. *F. racemosa* trees appear to exercise a degree of control over these processes, in both evolutionary and ecological time. Syconial architecture has developed such that fig ovules vary in profitability [19], [47], which selects for optimal foragers to focus on the more profitable inner ovules, at the cost of some fecundity [20]. Interference competition further reduces wasp fecundity, and the two effects ensure seed production over a broad range of wasp-hours. Fusing styles ensures that pollen reaches outer ovules, even if wasps try to pollinate only the stigmas that they oviposit into. *F. racemosa* trees also appear to be able to reduce the number of few-foundress syconia, and figs possibly also regulate lumen temperature and/or volume to limit wasp lifespans.

We expect that many of these phenomena will be found to stabilize the mutualism in other *Ficus* species, but with two important caveats. Firstly, *F. racemosa* is almost certainly derived recently from gynodioecious ancestors [29], meaning that the conflict-resolution mechanisms documented here have probably evolved independently from those in the large lineages of monoecious figs in other sections of the genus *Ficus*. Secondly, *F. racemosa* exhibits large syconia for the genus, so the problem of insufficient wasps is especially important in this species.

### Host control and its limits

It is arguable that host control in figs extends beyond even the mechanisms outlined above. Note that from the fitness standpoint of a fig tree producing wasps (‘donor’ trees), the ideal female wasp offspring disperses to a recipient tree, deposits pollen, and fails to lay a single egg, allowing all pollinated ovules to develop into seeds. The most fundamental way to achieve this is to reduce ovule size, which reduces the size of wasp offspring, and thus reduces egg loads, and possibly, lifespans [66], [67]. A secondary consequence could be that recipient trees are given increased control of arrived foundresses, if smaller wasps are more susceptible to heat stress, allowing figs to avoid over-exploitation using the mechanisms outlined above. We therefore hypothesize that one reason for the generally small size of fig seeds is selection for small wasp size.

There are, however, countervailing selection pressures that promote the survival of wasps after arrival, and thus, that maintain the mutualism: larger seeds are likely to be more viable, and wasps must be large enough to survive dispersal [19], [47]. Also important in this system is active pollination behavior in foundresses, which is a derived trait [54], [68] and counteracts selection on figs to reduce wasp size. With active pollination, seed production is parceled out with egg deposition, which creates a positive correlation between wasp lifetime and seed production, at least up to a point (Figure 1).

The fact that each wasp larva feeds on only one seed seems to be the fundamental explanation for how fig plants control the relationship, an asymmetry in power first suggested for figs by Herre [25]. The fig host can control the physiological parameters of its wasps, and thus can produce stressful environments when symbionts threaten over-exploitation, an outcome that is mechanistically indistinguishable from a suite of host responses to pathogenic symbionts, such as infection-induced fever in humans [69]. We suggest that this mode of evolution may play an important, but so far underappreciated, role in promoting the evolution and maintenance of mutualistic symbiosis.

## Materials and Methods

### Study species

The monoecious fig tree *Ficus racemosa* L. is distributed from India to Australia [70]. It can grow up to 30 m in height and produces large numbers of cauliflorous syconia. In primary forest it often occur in clusters of 5 to 10 individuals [71], typically near (semi-)permanent water (J. Cook, personal observation). At least in China, production of syconia is typically highest during the summer. Syconia typically complete their cycle (see [27]) in two to three months in the warm, rainy season and in three to four months during the winter. *F. racemosa* is actively pollinated by *Ceratosolen fusciceps* (Agaoninae). Active pollination means that the foundresses within receptive syconia exhibit certain behaviors that are only associated with the transfer of pollen from their pollen pockets to the floral stigmas.

### Study site

The study was performed at the Xishuangbanna Botanical Garden (N 21.9238, E 101.2511, alt ∼600 m above sea level) in southern Yunnan, China. The climate is subtropical with a rainy season (∼80% of annual rainfall) from May to October. Most of the sampled syconia were from a grove of trees (“Cluster A”) located in a ∼1 km^2^ forest fragment on the grounds of the botanical garden, and from a group of independent trees lining a local river and road (“Area B”). Samples were supplemented with syconia from trees in a neighboring town and around nearby crop fields. All syconia into which foundresses entered naturally are referred to as having been collected from the ‘wild,’ to differentiate them from syconia into which wasps were experimentally introduced.

### Galling, seed, season, and foundress number data

#### Wild-collected syconia

Data were collected from both wild and experimental syconia. The wild dataset pools various collections made from 1999 to 2005 covering all months of the year. A total of 251 of these syconia (n_”Cluster A”_ = 184, n_”Area B”_ = 37, n_all other sites_ = 30) were collected just prior to D-phase (wasp emergence phase [23]), and the dead foundress bodies (*NF*) in each syconium were counted. Syconia were collected just prior to D-phase because wasp offspring had matured sufficiently to be identified, but had not yet left the syconium.

#### Experimental-introduction syconia

The tunnel through which foundresses enter syconia, the ostiole, stays open from several hours to multiple days (see ***Density-dependent ostiole closure***). As a consequence, wild syconia typically contain foundresses that have entered at different times. Collections from wild syconia thus cannot give us information on the degree of temporal overlap of foundresses during oviposition (the fig's B-phase), which we hypothesize can affect oviposition success.

We therefore also present data from four experiments in which pre-determined numbers of foundresses were introduced into receptive syconia (B-phase) and then allowed to mature until they reached D-phase. In the first three experiments, introductions were conducted in the summer, between March and April 2007 and again in July 2008, on three different and widely separated trees.

On each of these trees, two different introduction schedules were followed: (1) short-interval (‘consecutive introductions’) and (2) long-interval (‘staggered introductions’). On two trees, 1, 2, 3, 5, 7 or 9 foundresses were introduced using the consecutive introduction schedule, and 2, 3, 5, 7 or 9 foundresses were introduced following the staggered-introduction schedule. (The 3-foundress, staggered-introduction treatment was omitted on one of the two trees.) Sample sizes ranged from 21 to 23 syconia for each combination of foundress number and interval schedule. On the third tree, we used only 9 foundresses for the consecutive (n = 22 syconia) and staggered (n = 23) introductions. Total sample size was 470 syconia.

In the consecutive-introduction treatment, all foundresses were introduced within a 30-min time window. In the staggered-introduction treatment, foundresses were introduced over 33 hours, using the following schedule: 2 foundresses at 09:00, day one; 2 at 15:00, day one; 2 at 09:00, day two; 2 at 15:00, day two; and 1 at 18:00, day two. Of course, only when nine foundresses were introduced did we follow the entire schedule. In the 2-wasp, staggered treatment, one wasp was introduced at 9am on day one, and the second was introduced at 9am on day two. After the introductions, syconia were bagged with organdy cloth to prevent attack by parasitic wasps that oviposit from outside the syconium.

Treatments were performed over the course of single, asynchronously produced fruit crops. Entire racemes were used for each treatment because tags on individual syconia could occasionally be removed by passers-by. Before syconia on a raceme became receptive, they were bagged to prevent wasp entry. Foundresses were then collected using an insect net from the air surrounding receptive syconia. One by one, each syconium was debagged. The net containing the caught foundresses was then held over the entrance of a newly exposed, receptive syconium until the required number of foundresses had entered. All syconia were then re-bagged.

In short, our experimental design includes a continuous treatment, foundress number, crossed with a categorical treatment, consecutive vs. staggered introduction schedules. Both types of introductions were conducted on each of the three trees. These data are used for two purposes: to test for the effect of temporal overlap in foundress galling success and to compare with the summer wild-collection syconia (consecutive introduction only).

In the fourth experiment, introductions were conducted in the winter (Nov 1999 to Jan 2000) and comprised only consecutive introductions of 2, 5 and 8 foundresses (n = 13, 25, and 17 foundresses, respectively) on one tree. This dataset is used only to compare with the winter wild-collection syconia. Wild syconia contain between 1 and 78 foundresses, with 52% containing from 1 to 9 foundresses. Preliminary analysis of the wild data showed that most of the observed variation in galling and pollination was represented in syconia with less than 10 foundresses. Experimental introductions for higher numbers of foundresses were thus not performed.

For both the wild and experimental datasets, after maturity (D-phase), syconia were dissected and scored for the number of galls (flowers containing a pollinator wasp larva), seeds, and vacant flowers, which can be distinguished visually. Each syconium was cut vertically into eight equal slices passing through the ostiole. Two or three slices were haphazardly selected to count galls, seeds, and all vacant flowers. For the remaining 5 or 6 slices, only galls and seeds were counted, due to the difficulty of counting vacant flowers. The percentage of developed flowers (galls + seeds) per syconium is thus based on a sub-sample of each syconium, while the ratio of galls to seeds is based on the entire syconium.

Statistical analyses of the wild-collected and the experimental, consecutive-introduction syconia were conducted with the *nlme* 3.1–90 package in *R* 2.8.0 [72]. We used a random-factor, general linear model to test for the effects of season (categorical, winter vs. summer) and foundress number (continuous) on the frequency of galls: galls/(galls + seeds). Tree was included as a random factor. In the wild-collection data, some of which we collected from unpublished data by colleagues, the majority of syconia could not be identified to a particular tree, and these syconia (n = 164) were assigned to a single, notional tree and included as the seventh tree out of seven.

We also used random-factor GLMs to test for the effects of introduction schedule (categorical, consecutive vs. staggered) and foundress number on the frequency of galls in the experimental-introduction syconia.

### Environmental effects on foundress lifespans

Thirty D-phase syconia were collected from Site “B” trees in April 2004. These were cut open, and a total of 5684 total foundresses was removed. These foundresses were assigned to six experimental treatments, each with 8 replicates, comprising three temperature levels (18°C, 22°C, 30°C) crossed with two humidity levels (100% and 70%). Each experimental treatment began at 09:00. Immediately after cutting open a mature syconium, the emerged foundresses were put into 50 ml Erlenmeyer flasks and sealed with organdy cloth. This ensured minimal variation in elapsed lifetimes between the pollinators, but the numbers of foundresses per flask necessarily varied. The 48 flasks (8 replicates by 6 treatments) were then placed into a darkened CLIMACELL climate chamber (MMM Medcenter, Munich) to regulate temperature and humidity. Foundresses were observed at 6, 12, 24, 30, 36 and 48 hours after entry into the flasks. At each observation, the numbers of dead foundresses were recorded.

Statistical analyses were performed using the R package *survival* 2.3.0. To estimate seasonal differences in mean expected lifespan, we used the *survreg* function to fit a parametric model to the right-censored survivorship data. The continuous explanatory variables were temperature and humidity. In the results presented here, the hazard distribution used is exponential (i.e., constant hazard across all age classes), but the use of accelerated hazard with age (Weibull and Rayleigh distributions) did not change the overall outcome (data not shown). We also tested for differences in survival between experimental treatments with the non-parametric Cox proportional hazard model, using temperature, humidity, and starting density as explanatory variables.

Note that the utility of these experimental results for interpreting the effects of season depends on the assumption that syconium-internal temperatures reflect external environmental temperatures, which is reasonable for plants, and has been shown directly for D-phase syconia in *Ficus yopoensis* [58].

### Estimating oviposition lifespans and ovule selectivity

The numbers of galls and seeds are determined by two factors: how long the wasps oviposit for (time budget) and which ovules are selected for oviposition (selectivity). Unfortunately, oviposition lifespans and ovule selectivity are not directly observable, nor are they simple to infer from raw counts of galls and seeds. For example, if two foundresses produce three times as many galls as one foundress, it may be because they lived longer. Alternatively, foundresses may have been less choosy, even at the cost of producing offspring of lower average viability [19]. Even if we assume that there is no difference in levels of choosiness, we can only say that each of the two foundresses galled for longer than the sole foundress. As to how much longer, we have little idea, in part because galling rate declines as the frequency of un-galled ovules declines [20].

To estimate oviposition lifespans and ovule selectivity, we fitted a simulation model of oviposition to the seed and gall data. Oviposition lifespans were measured in “effective mm” of flower styles probed. If a foundress probes a 1 mm-long style, that is 1 mm of oviposition lifespan. If she also oviposits down that 1 mm of style, that counts as a further *k*×1 mm of lifespan, where *k* is the ratio of oviposition time to probe time. For the analyses presented here, *k* is merely a scaling parameter. We present the results for *k* = 10. Repeat runs using *k* = 3 and *k* = 25 yielded results less than 1% different (data not shown). Ovule selectivity is measured as the maximum style length beyond which foundresses choose not to oviposit.

In each run of the model, a given number of wasps all search randomly within a single syconium for egg-free ovules. All foundresses are given the same lifespan in terms of “effective mm,” and the same degree of selectivity in terms of a maximum style length, such that they are willing to oviposit in all ovules with styles less than this length. After oviposition, they also deposit pollen on the stigma. In *F. racemosa*, groups of styles are fused (for details see paragraph below), so in our model, the pollen is distributed to all the styles that are fused to the focal style, and seeds are produced in the attached ovules, as long as they do not subsequently receive an egg. The model makes no assumptions concerning the optimality of oviposition behavior (Figure S1).

Style lengths are generated from a normal distribution [43] with a mean of 2.09 mm and standard deviation 0.516 mm, where these parameters were estimated from 335 measured styles (R. Wang, unpublished data). Style fusions are simulated in the model using the observation that longer styles are always fused to shorter styles (R. Wang, personal observation). We assume that the 2/3 of styles that are longest are fused to the 1/3 of styles that are shortest. For the shortest 1/3, the frequency of fusions is linearly related to style rank (shorter styles being ranked higher). The choice of 1/3 as the switch point follows from the observation that seeds start to diminish once galling exceeds 30–40% (see **Results**).

The estimation of the lifespan and ovule selectivity parameters from each syconium's seed and gall data is done in two stages. First, we define the parameter space by running the model over a range of lifetime and selectivity parameter pairs (Figure S2). This reveals a 1∶1 relationship between a pair of gall and seed data points, and a pair of lifespan and selectivity parameters. Because of this 1∶1 relationship, we can interpolate, using cubic splines, within the model outputs illustrated in Figure S2, to estimate oviposition lifespan and selectivity for each measured syconium.

### Density-dependent ostiole closure

Each receptive *Ficus* syconium has a small, bract-filled opening, the ostiole, through which fig wasps pass into the central lumen [24], [35]. It is from the lumen that the wasps pollinate and oviposit. We tested the hypothesis that ostioles close more rapidly the more foundresses enter initially. For this experiment, in November and December of 2001, the lower trunks of two trees were wrapped with organdy cloth in order to prevent wasp entry. When the syconia on a raceme became receptive (B-phase), all syconia on that raceme were assigned to one of five treatment levels: the experimental introduction of 1, 3, 5, 7, or 9 foundresses, which had been first caught with an insect net near to the trees. Throughout, care was taken to use syconia of a similar size. Wasps were introduced following the same protocol as above (see **Galling, pollination, and foundress number data**).

Pilot experiments were used to fix the approximate temporal ranges of ostiole closure times. Once these were determined, more detailed bioassays were performed. For example, in the 9-wasp treatment, after 2 hours, ostiole closure was tested by using a translucent organdy bag to enclose a test syconium with a newly-caught wasp. Usually the wasp walked toward the entrance, but occasionally, a drinking straw was used to hold a wasp against the ostiole, so the experimenter could gently blow the wasp towards the ostiole. Either way, once the wasp was at an open ostiole, it entered, usually taking under a minute. Foundresses vary enough in size that size can be distinguished visually, and we used only larger wasps. Thus, if a large-bodied wasp was able to enter a fig, that fig was no longer counted (because it now had, for example, 9+1 wasps). A new fig from the same treatment level was tested between 30–60 minutes later. If a large-bodied wasp could not enter but a small-bodied wasp could, or could get at least halfway in, then that time was counted as the onset of ostiole closure. If even a small wasp could not enter, then the ostiole was counted as entirely closed, and the closing time scored as occurring halfway between this census time and the last.

### Estimating the effect of ostiole closure on foundress number distribution

We estimated the effect of ostiole closure on foundress numbers as follows. We used the *nlm* function in *R* to fit a logistic relationship between foundress number and time to ostiole closure. For more than 9 foundresses we used the 9-foundress closing time. (Preliminary experiments have not found that additional wasps further reduce ostiole closure time, R. Wang, unpublished data.)

Additionally, we used the data on foundress numbers inside wild syconia. Foundress numbers for our survey of wild syconia vary with season, averaging 10.6 (n = 81) in the winter, and 16.9 (n = 112) in the summer. This dataset excludes syconia with foundress number (*NF*)  = 0 (n = 33) and a small number of syconia (n = 26) that were collected to boost the sample size of low-*NF* syconia in the summer. There were significantly more foundresses per syconium in the summer than in the winter (*p* = 0.001, t_80_ = 3.3).

From the ostiole closure times and the frequency distribution of foundresses inside syconia, we infer the distribution of wasps arriving at syconia as follows. We assume a constant arrival probability for wasps at syconia, giving us an exponential distribution for the intervals between wasp arrivals: Prob(interval length  =  x)  =  αe^−αx^. We further assume wasps arrive in groups, the size of which follows a geometric distribution: Prob(group size  =  k)  =  *(1−β)^k^β*.

For a wide range of geometric distributions (β_i_ = 0.05, 0.055, 0.06, …, 0.15), we iteratively simulate 1000 syconia until we find α_I_, defining the intervals between wasp arrivals, that yields the best match to the observed distributions of foundresses per syconium. This gives us a range of parameter pairs (α_1..n_, β_1..n_), from which we select the pair that best matches the observed distributions of foundress numbers. To find a best match, the observed foundress numbers and the simulated foundress numbers are binned in ranges: 1–3, 4–6, >6, with the best match being that which minimizes the squared deviations between observed and simulated distributions.

For each season, we thus have a simulated distribution of foundress numbers, derived from an exponential interval distribution and a geometric group-size distribution that matches the observed distributions. To see what would happen in the absence of density-dependent ostiole closure, we assume ostioles stay open until all arriving wasp groups have been assigned at random to a syconium. The number of syconia is chosen so that the seasonal means, for foundress number per syconium, match the observed data.

## Supporting Information

Figure S1 Flow diagram summarizing the oviposition simulation model
(1.70 MB TIF)

Figure S2 Model-generated relationships between galling and pollination. Each line is the trajectory assuming a different ‘maximum style length for galling.’ Here, we show four trajectories, with ovules at the end of styles over 1.75, 2.0, 2.25 and 2.5 mm being deemed too long to be galled, respectively. Thus, the selectivity parameter determines the gradient of the trajectory. The effective lifespan parameter determines the distance from the origin. For higher values of the selectivity parameter (i.e., longer maximum style length), the distance from the origin plateaus at a value on the line y = −x, while for low values the plateau falls short of this because some style clusters contain no styles short enough to be galled in, and thus, galls within these clusters are neither galled nor pollinated. In summary, each point on the graph corresponds to a unique combination of seeds and galls and thus, to a unique pair of effective lifespan and style selectivity.
(1.19 MB TIF)

Figure S3 Lifespan estimates by month, based on the model fitted to laboratory lifespan data. (Mean lifetime  = 4.0−0.13*Temp + (0.017+0.0004*Temp)*Humidity). All parameters are significant at p<0.001.
(1.35 MB TIF)

Text S1 (0.04 MB DOC)

Text S2 (0.04 MB DOC)

Text S3 (0.03 MB DOC)
