# Supplementary figures and images for: Interference Competition and High Temperatures Reduce the Virulence of Fig Wasps and Stabilize a Fig-Wasp Mutualism
Source: PLoS One. 2009 Nov 12;4(11):e7802. doi: 10.1371/journal.pone.0007802 (PMC2771911; doi:10.1371/journal.pone.0007802)

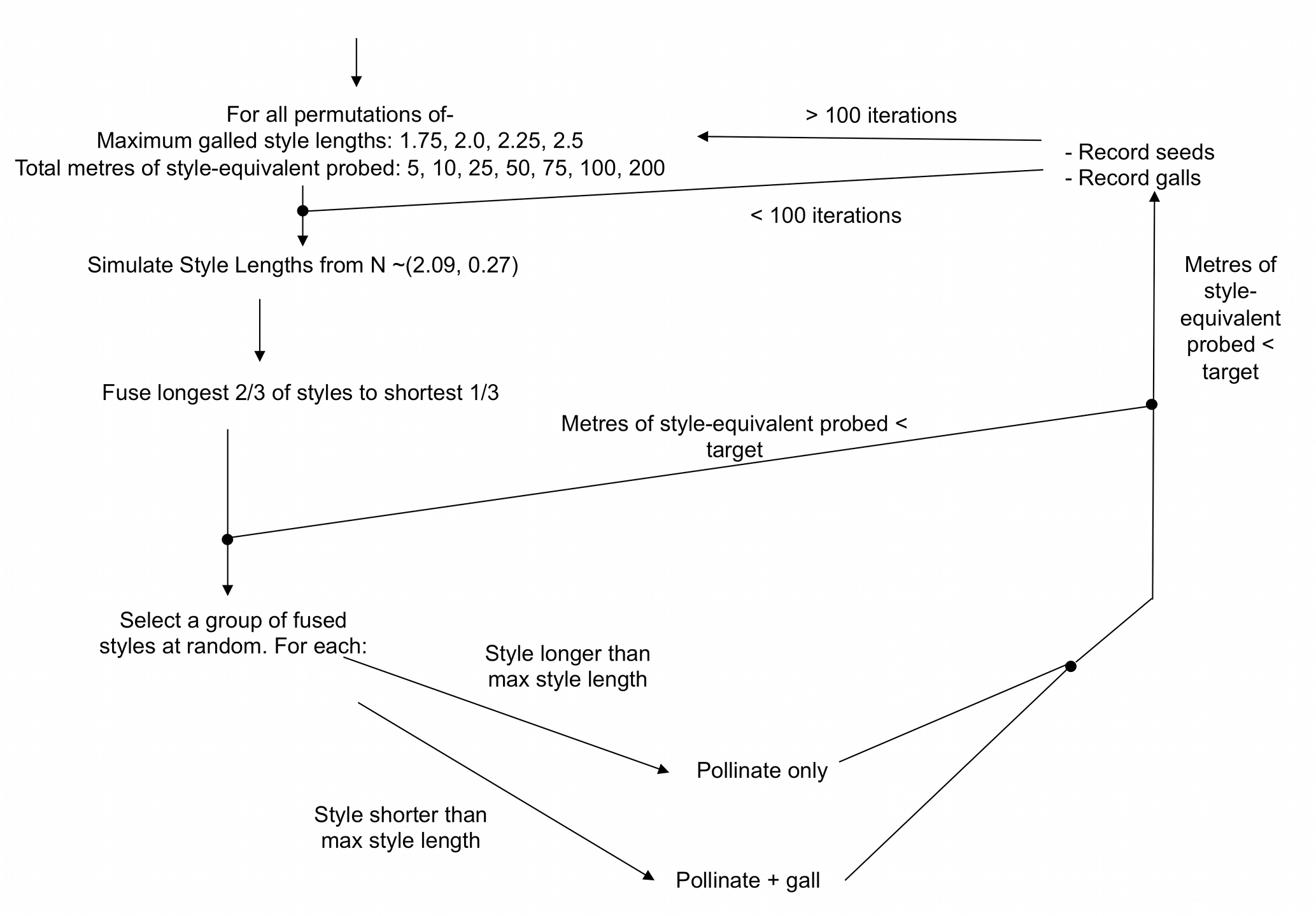

Supplement: Figure S1 — Flow diagram summarizing the oviposition simulation model (1.70 MB TIF) [file pone.0007802.s001.tif]

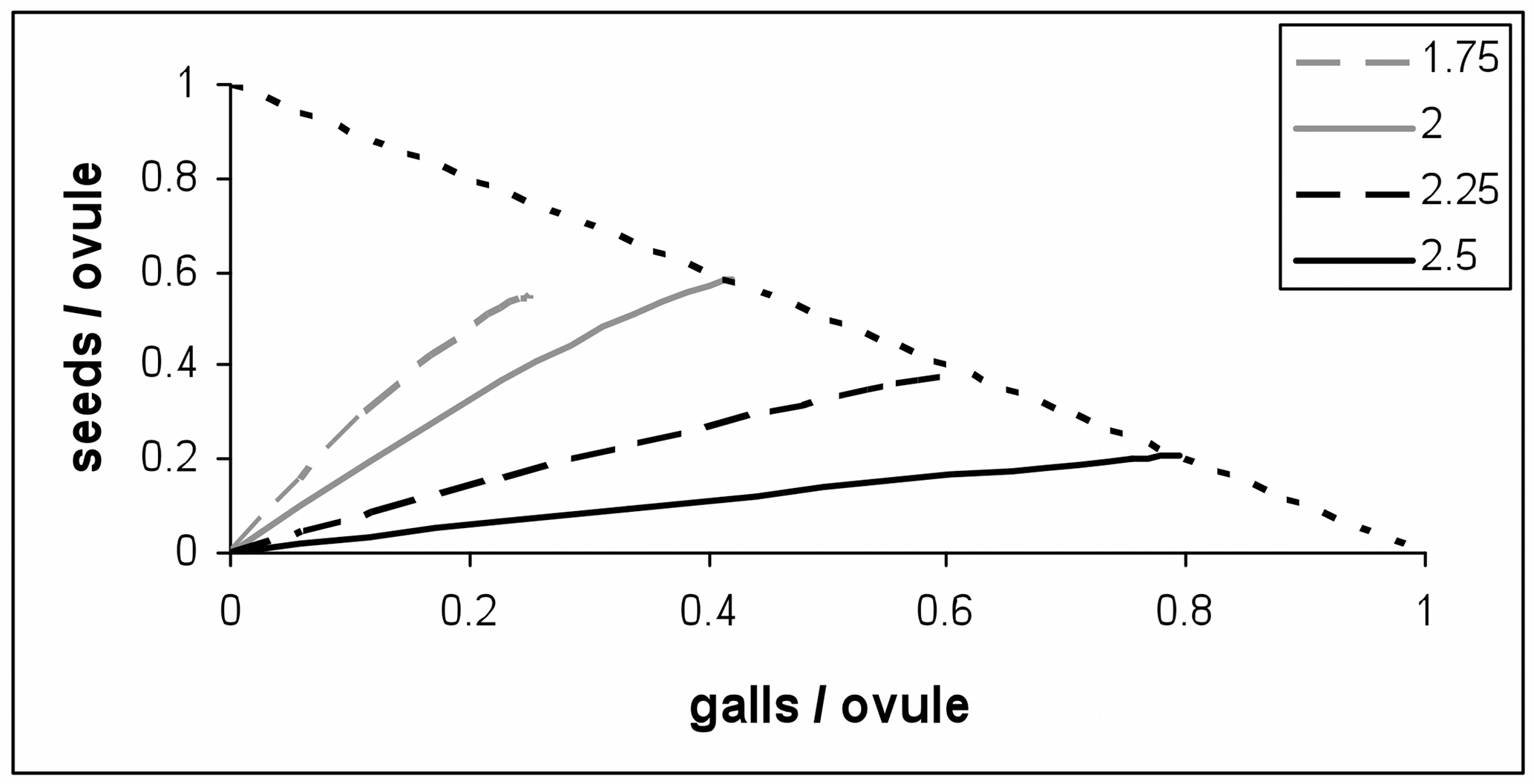

Supplement: Figure S2 — Model-generated relationships between galling and pollination. Each line is the trajectory assuming a different ‘maximum style length for galling.’ Here, we show four trajectories, with ovules at the end of styles over 1.75, 2.0, 2.25 and 2.5 mm being deemed too long to be galled, respectively. Thus, the selectivity parameter determines the gradient of the trajectory. The effective lifespan parameter determines the distance from the origin. For higher values of the selectivity parameter (i.e., longer maximum style length), the distance from the origin plateaus at a value on the line y = −x, while for low values the plateau falls short of this because some style clusters contain no styles short enough to be galled in, and thus, galls within these clusters are neither galled nor pollinated. In summary, each point on the graph corresponds to a unique combination of seeds and galls and thus, to a unique pair of effective lifespan and style selectivity. (1.19 MB TIF) [file pone.0007802.s002.tif]

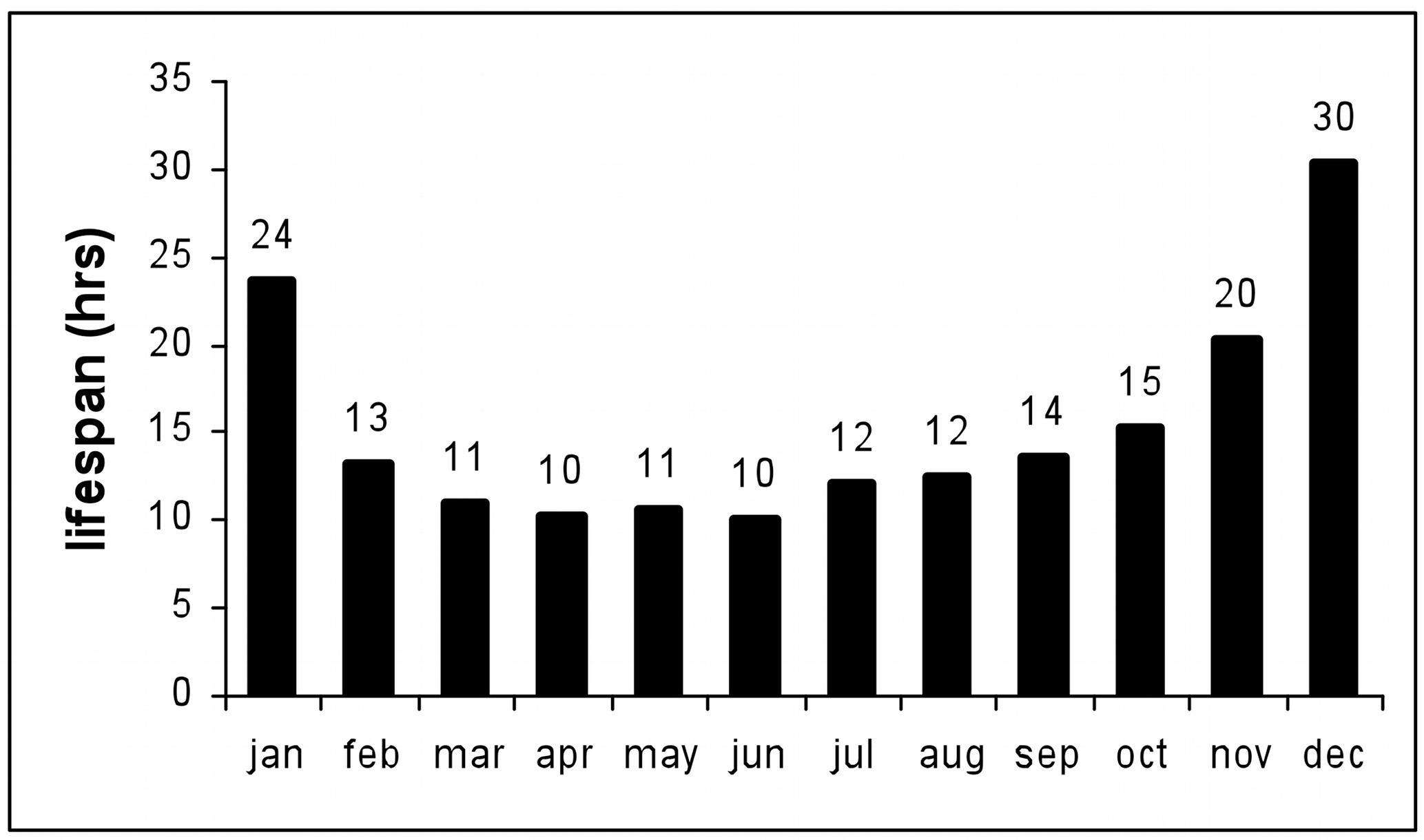

Supplement: Figure S3 — Lifespan estimates by month, based on the model fitted to laboratory lifespan data. (Mean lifetime = 4.0−0.13*Temp + (0.017+0.0004*Temp)*Humidity). All parameters are significant at p<0.001. (1.35 MB TIF) [file pone.0007802.s003.tif]
