# Supplementary material for: Interference Competition and High Temperatures Reduce the Virulence of Fig Wasps and Stabilize a Fig-Wasp Mutualism
Source: PLoS One. 2009 Nov 12;4(11):e7802. doi: 10.1371/journal.pone.0007802 (PMC2771911; doi:10.1371/journal.pone.0007802)
Supplement: Text S1 — (0.04 MB DOC) [file pone.0007802.s004.doc]

**Text S1. The effects of season and foundress number on the percentage of galled ovules: background statistics.** We present two analyses of the galling, seed, season and foundress data (Figure 1). The response variable is GALLPCT = galls/(galls + seeds), and we have one categorical predictor (SEASON), one continuous predictor (FOUNDRESSES), and one random variable (TREEID). We use a random-effect general linear model to test for the significance and direction of the FOUNDRESS by SEASON interaction effect in both the experimental and wild datasets. The following is the raw R output from our analyses.

**Experimental dataset, Random effects GLM**

b1<-lme(fixed=GALLPCT~FOUNDRESSES:factor(SEASON), random= ~ 1 | factor(TREEID), data=y);

> summary(b1);

Linear mixed-effects model fit by REML

Data: y

AIC BIC logLik

-722.0589 -703.296 366.0294

Random effects:

Formula: ~1 | factor(TREEID)

(Intercept) Residual

StdDev: 0.3094681 0.07104421

Fixed effects: GALLPCT ~ FOUNDRESSES:factor(SEASON)

Value Std.Error DF t-value p-value

(Intercept) 0.4388573 0.15509230 312 2.829652 0.005

FOUNDRESSES:factor(SEASON)summer 0.0118866 0.00162122 312 7.331895 0.000

FOUNDRESSES:factor(SEASON)winter -0.0447949 0.00433603 312 -10.330871 0.000

Correlation:

(Intr) FOUNDRESSES:fctr(SEASON)s

FOUNDRESSES:factor(SEASON)summer -0.047

FOUNDRESSES:factor(SEASON)winter -0.037 0.002

Standardized Within-Group Residuals:

Min Q1 Med Q3 Max

-3.99289519 -0.52446483 -0.01252940 0.60314740 3.52877162

Number of Observations: 318

Number of Groups: 4

**Wild dataset, Random effects GLM**

b1<-lme(fixed=GALLPCT~FOUNDRESSES:factor(SEASON), random= ~ 1 | factor(TREEID), data=y); summary(b1)

Linear mixed-effects model fit by REML

Data: y

AIC BIC logLik

-215.2006 -198.3242 112.6003

Random effects:

Formula: ~1 | factor(TREEID)

(Intercept) Residual

StdDev: 0.05111591 0.1331995

Fixed effects: GALLPCT ~ FOUNDRESSES:factor(SEASON)

Value Std.Error DF t-value p-value

(Intercept) 0.5788721 0.025326787 210 22.856122 0.0000

FOUNDRESSES:factor(SEASON)summer 0.0001106 0.000715822 210 0.154552 0.8773

FOUNDRESSES:factor(SEASON)winter 0.0031705 0.001122685 210 2.824066 0.0052

Correlation:

(Intr) FOUNDRESSES:fctr(SEASON)s

FOUNDRESSES:factor(SEASON)summer -0.304

FOUNDRESSES:factor(SEASON)winter -0.273 0.305

Standardized Within-Group Residuals:

Min Q1 Med Q3 Max

-2.25929822 -0.64298768 -0.09968488 0.61631330 2.53038949

Number of Observations: 219

Number of Groups: 7
