# Supplementary material for: Interference Competition and High Temperatures Reduce the Virulence of Fig Wasps and Stabilize a Fig-Wasp Mutualism
Source: PLoS One. 2009 Nov 12;4(11):e7802. doi: 10.1371/journal.pone.0007802 (PMC2771911; doi:10.1371/journal.pone.0007802)
Supplement: Text S2 — (0.04 MB DOC) [file pone.0007802.s005.doc]

**Text S2. Experimental evidence that wasps reduce each others’ oviposition rates: background statistics.**  We present fixed-effects and random-effects general linear models of the experimental introduction experiment (Figure 5). Only the best-fitting models are presented. Other models included syconial diameter, but this predictor was not retained or produced worse-fitting models. The response variable is GALLPCTTOT = galls/(galls + seeds + empty ovules). We have categorical predictor (TREATMENT = consecutive vs. staggered), one continuous predictor (FOUNDRESSES), and one random or categorical variable, depending on the model used (TREEID). Similar results are found when the response variable is GALLPCT = galls/(galls + seeds), but the variance explained is lower (results not shown). The following is the raw R output from our analyses.

**Fixed-effects model, with two categorical factors, Treatment and TreeID (n = 3), and one continuous variable, Foundresses.**

**Main effects only**

a<-lm(Gallpcttot~Foundresses+factor(Treatment)+factor(TreeID),data=y5); summary(a); anova(a);

Call:

lm(formula = Gallpcttot ~ Foundresses + factor(Treatment) + factor(TreeID),

data = y5)

Residuals:

Min 1Q Median 3Q Max

-0.188203 -0.045768 0.001977 0.048019 0.249877

Coefficients:

Estimate Std. Error t value Pr(>|t|)

(Intercept) 0.056279 0.008495 6.625 9.63e-11 ***

Foundresses 0.027565 0.001322 20.855 < 2e-16 ***

factor(Treatment)staggered 0.097876 0.007014 13.955 < 2e-16 ***

factor(TreeID)11 0.117880 0.013362 8.822 < 2e-16 ***

factor(TreeID)13 0.008917 0.007203 1.238 0.216

---

Signif. codes: 0 ‘***’ 0.001 ‘**’ 0.01 ‘*’ 0.05 ‘.’ 0.1 ‘ ’ 1

Residual standard error: 0.07414 on 465 degrees of freedom

Multiple R-squared: 0.7131, Adjusted R-squared: 0.7106

F-statistic: 288.9 on 4 and 465 DF, p-value: < 2.2e-16

Analysis of Variance Table

Response: Gallpcttot

Df Sum Sq Mean Sq F value Pr(>F)

Foundresses 1 4.8661 4.8661 885.198 < 2.2e-16 ***

factor(Treatment) 1 1.0527 1.0527 191.497 < 2.2e-16 ***

factor(TreeID) 2 0.4348 0.2174 39.545 < 2.2e-16 ***

Residuals 465 2.5562 0.0055

---

Signif. codes: 0 ‘***’ 0.001 ‘**’ 0.01 ‘*’ 0.05 ‘.’ 0.1 ‘ ’ 1

**Adding an interaction effect.**

> a<-lm(gallpcttot~Foundresses+factor(Treatment)+Foundresses*factor(Treatment)+factor(TreeID),data=Dataset); summary(a); anova(a);

Call:

lm(formula = gallpcttot ~ Foundresses + factor(Treatment) + Foundresses *

factor(Treatment) + factor(TreeID), data = Dataset)

Residuals:

Min 1Q Median 3Q Max

-0.201965 -0.046419 -0.007168 0.042819 0.210123

Coefficients:

Estimate Std. Error t value

(Intercept) 0.097566 0.008551 11.410

Foundresses 0.018664 0.001451 12.866

factor(Treatment)[T.staggered] -0.032215 0.013734 -2.346

factor(TreeID)[T.11] 0.113165 0.011997 9.433

factor(TreeID)[T.13] 0.013610 0.006478 2.101

Foundresses:factor(Treatment)[T.staggered] 0.023710 0.002225 10.657

Pr(>|t|)

(Intercept) <2e-16 ***

Foundresses <2e-16 ***

factor(Treatment)[T.staggered] 0.0194 *

factor(TreeID)[T.11] <2e-16 ***

factor(TreeID)[T.13] 0.0362 *

Foundresses:factor(Treatment)[T.staggered] <2e-16 ***

---

Signif. codes: 0 '***' 0.001 '**' 0.01 '*' 0.05 '.' 0.1 ' ' 1

Residual standard error: 0.06653 on 464 degrees of freedom

Multiple R-squared: 0.7695, Adjusted R-squared: 0.767

F-statistic: 309.8 on 5 and 464 DF, p-value: < 2.2e-16

Analysis of Variance Table

Response: gallpcttot

Df Sum Sq Mean Sq F value Pr(>F)

Foundresses 1 4.8661 4.8661 1099.488 < 2.2e-16 ***

factor(Treatment) 1 1.0527 1.0527 237.855 < 2.2e-16 ***

factor(TreeID) 2 0.4348 0.2174 49.119 < 2.2e-16 ***

Foundresses:factor(Treatment) 1 0.5026 0.5026 113.568 < 2.2e-16 ***

Residuals 464 2.0536 0.0044

---

Signif. codes: 0 '***' 0.001 '**' 0.01 '*' 0.05 '.' 0.1 ' ' 1

**Random-effects model as analyzed in R 2.8.0, with one categorical factor, Treatment, one continuous variable, Foundresses, and one random factor, TreeID (n = 3).**

**Main effects only**

b3<-lme(fixed=Gallpcttot~Foundresses+factor(Treatment), random= ~ 1 | factor(TreeID), data=y5);

> summary(b3);

Linear mixed-effects model fit by REML

Data: y5

AIC BIC logLik

-1066.857 -1046.126 538.4286

Random effects:

Formula: ~1 | factor(TreeID)

(Intercept) Residual

StdDev: 0.06445818 0.07414454

Fixed effects: Gallpcttot ~ Foundresses + factor(Treatment)

Value Std.Error DF t-value p-value

(Intercept) 0.09720147 0.03838772 465 2.532098 0.0117

Foundresses 0.02768865 0.00131892 465 20.993502 0.0000

factor(Treatment)staggered 0.09784120 0.00701386 465 13.949687 0.0000

Correlation:

(Intr) Fndrss

Foundresses -0.200

factor(Treatment)staggered -0.047 -0.175

**Adding an interaction effect**

> b3<-lme(fixed=**gallpcttot**~Foundresses+factor(Treatment)+Foundresses*factor(Treatment), random= ~ 1 | factor(TreeID), data=Dataset);summary(b3)

Linear mixed-effects model fit by REML

Data: Dataset

AIC BIC logLik

-1156.525 -1131.660 584.2627

Random effects:

Formula: ~1 | factor(TreeID)

(Intercept) Residual

StdDev: 0.06078388 0.06652781

Fixed effects: gallpcttot ~ Foundresses + factor(Treatment) + Foundresses * factor(Treatment)

Value Std.Error DF t-value p-value

(Intercept) 0.13872721 0.03630524 464 3.821135 0.0002

Foundresses 0.01875894 0.00144908 464 12.945392 0.0000

factor(Treatment)[T.staggered] -0.03238427 0.01373338 464 -2.358070 0.0188

Foundresses:factor(Treatment)[T.staggered] 0.02373535 0.00222475 464 10.668770 0.0000

Correlation:

(Intr) Fndrss f(T)[T

Foundresses -0.217

factor(Treatment)[T.staggered] -0.115 0.447

Foundresses:factor(Treatment)[T.staggered] 0.107 -0.577 -0.889

Standardized Within-Group Residuals:

Min Q1 Med Q3 Max

-3.0425379 -0.7009593 -0.1048523 0.6429142 3.1863030

Number of Observations: 470

Number of Groups: 3
