# Supplementary material for: Interference Competition and High Temperatures Reduce the Virulence of Fig Wasps and Stabilize a Fig-Wasp Mutualism
Source: PLoS One. 2009 Nov 12;4(11):e7802. doi: 10.1371/journal.pone.0007802 (PMC2771911; doi:10.1371/journal.pone.0007802)
Supplement: Text S3 — (0.03 MB DOC) [file pone.0007802.s006.doc]

**Text S3. A model of syconial warming from fig wasps’ metabolic heat.** We take a syconium to be a sphere of diameter 4 cm, comprising a central spherical volume of air (diameter 1 cm) surrounded by a 1.5 cm thick water jacket. The mass-specific resting heat production of fully fed adult *Drosophila* flies at 25°C is 17.3 µW/mg [65]. Assuming the same figure for fig wasps, estimating their weight at 1 mg and using the thermal conductivity of water (0.6 W/m. °C), we approximate heat loss through the fig as a linear heat flow problem, and find the temperature gradient between the lumen and the outside of the fig to be only 0.00016°C per wasp.
